# Distribution of *Rickettsia rickettsii* in ovary cells of *Rhipicephalus sanguineus* (Latreille1806) (Acari: Ixodidae)

**DOI:** 10.1186/1756-3305-4-222

**Published:** 2011-11-25

**Authors:** Luís Flávio da Silva Costa, Pablo Henrique Nunes, João Fábio Soares, Marcelo Bahia Labruna, Maria Izabel Camargo-Mathias

**Affiliations:** 1 Departamento de Biologia, Instituto de Biociências UNESP - Rio Claro/SP, Brasil; 2 Laboratório de Doenças Parasitárias do Departamento de Medicina Veterinária Preventiva e Saúde Animal - VPS, USP - São Paulo, Brasil

**Keywords:** *Rickettsia rickettsii*, *Rhipicephalus sanguineus*, Brazilian Spotted Fever, ovary, histology

## Abstract

**Background:**

Considering the fact that the dog tick, *Rhipicephalus sanguineus*, has a great potential to become the vector of Brazilian Spotted Fever (BSF) for humans, the present study aimed to describe the distribution of the bacterium *Rickettsia rickettsii*, the etiological agent of BSF, in different regions of the ovaries of *R. sanguineus *using histological techniques. The ovaries were obtained from positive females confirmed by the hemolymph test and fed in the nymph stage on guinea pigs inoculated with *R. rickettsii*.

**Results:**

The results showed a general distribution of *R. rickettsii *in the ovary cells, being found in oocytes in all stages of development (I, II, III, IV and V) most commonly in the periphery of the oocyte and also in the cytoplasm of pedicel cells.

**Conclusions:**

The histological analysis of the ovaries of *R. sanguineus *infected females confirmed the presence of the bacterium, indicating that the infection can interfere negatively in the process of reproduction of the ticks, once alterations were detected both in the shape and cell structure of the oocytes which contained bacteria.

## Background

*Rickettsia rickettsii*, the etiological agent of Brazilian Spotted Fever (BSF) in Brazil and Rocky Mountain Spotted Fever in the United States [1] is an obligatory intracellular gram-negative bacterium which survives for a short time out of the host [2], being transmitted to humans and other animals by different species of ticks. These organisms are usually 0.8 to 2 μm long, with a diameter of 0.3 to 0.5 μm [3] and having a cellular wall formed by peptidoglycan and lipopolysaccharides [4]. The infection caused by rikettsiae is systemic in ticks as they multiply in the cytoplasm of the intestine, ovaries, salivary glands, Malpighian tubules cells and are also found in the hemolymph of the ectoparasite [5]. Following infection of the ovaries, transstadial and transovarial transmission of the pathogen can happen [6].

*Rickettsia rickettsii *is considered the most pathogenic species of rickettsia, being reported in Canada, United State, Mexico, Costa Rica, Panama, Brazil, Colombia and Argentina [7]. Many ticks are known as vectors of *R. rickettsia *in the world. In Brazil the known vectors are *Amblyomma cajennense*, tick vector in most of the endemic areas in the country and *A. aureolatum*, responsible for the transmission in some metropolitan areas of São Paulo [8,9]. Recent studies point to *R. sanguineus *as a possible vector of *R. rickettsii *for humans in some regions of Brazil. Moraes-Filho *et al*. [10] reported the presence of *R. sanguineus *positive for *R. rickettsii *in the metropolitan region of São Paulo and Cunha *et al*. [11] reported the same for Rio de Janeiro state and Pacheco *et al*. [12] in Juiz de Fora, Minas Gerais. Although *R. sanguineus *has not been confirmed as vector of spotted fever for humans in Brazil, it has been confirmed as a vector in the USA and Mexico [13] and has also been considered as a possible vector in Colombia [5]. Dantas-Torres *et al*. [14] and Louly *et al*. [15] reported human parasitism by *R. sanguineus *in the brazilian states of Pernambuco and Goiás, reinforcing the hypothesis that this tick can become a vector of *R. rickettsii *for humans in Brazil.

The *R. sanguineus *tick is originally from Africa and is commonly known as the "brown dog tick", with a wide geographic distribution [16]. This species was introduced into the urban environment by the domestic dog, which is considered its main host [17]. It is important to emphasize that *R. sanguineus *is the only species of tick that is considered an "urban plague" as it parasitizes dogs in urban and rural areas [18].

The ovary of the tick is located in the posterior third of the body, having a horseshoe shaped. The ovary of *R. sanguineus *is histologically classified as panoistic, with the lumen delimited by a delicate wall of small epithelial cells where the oocytes are fixed by the pedicel in all phases of development (stages I to V) [19].

Considering these facts, the aim of this study was to analyze through histological techniques the distribution of the bacterium *R. rickettsii *in the ovaries of fully-fed and semi-engorged *R. sanguineus *females, contributing to a better knowledge about the interaction between *R. rickettsii *and ticks in general.

## Results

### Confirmation of infection by *R. rickettsii*

In the first infestation (feeding of the nymphs), on the fifth day after the inoculation of the homogenate containing the bacterium, the guinea pigs presented with fever (temperature > 40°C) and one of them presented with a scrotal lesion and died on the ninth day. The animals of the control group did not present with a temperature increase during the whole infestation.

In the second infestation (feeding of adults) the three guinea pigs infested by adult ticks from the infected group of the first phase presented with fever (temperature > 40°C) and one of them presented with a scrotal lesion and died on the ninth day. The individuals of the control group did not present with a temperature increase during the whole infestation.

**Figure 1:**
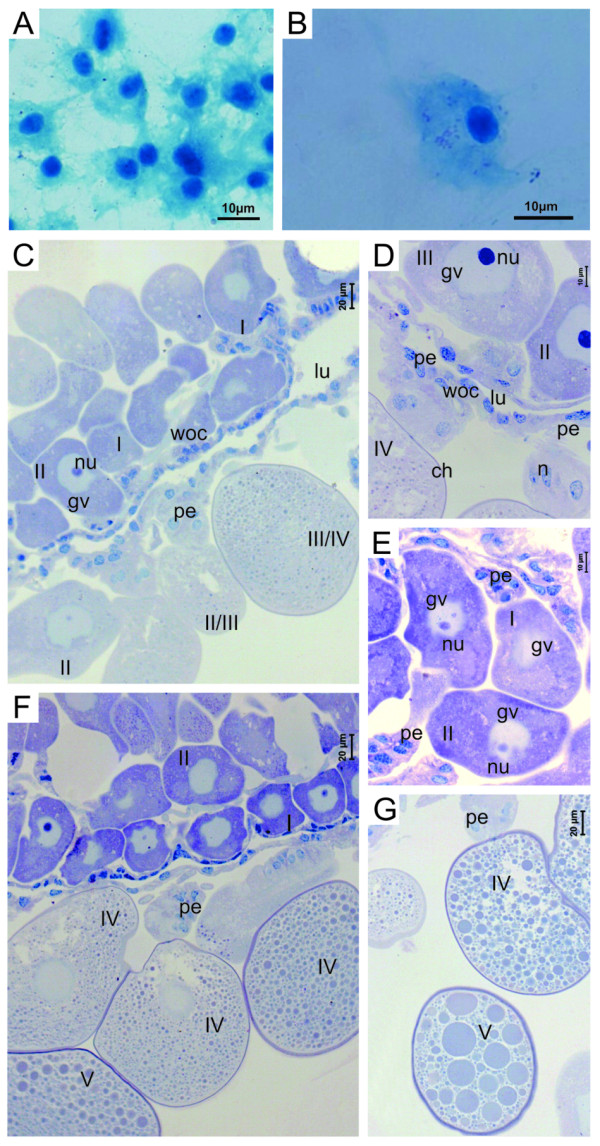
**Hemolymph cells and histological sections of *Rhipicephalus sanguineus *oocytes of control group**. (A) hemocytes with normal morphology stained using the Gimenez technique [28]. (B) Hemocyte with the presence of *R. rickettsii *stained using the Gimenez technique [28]. C, D, E, F and G, detail of oocytes in all stages of development (I, II, III, IV and V) using Giemsa staining. Ch, choriun; gv, germinal vesicle; lu, lumen; nu, nucleus; p, pedicel; woc, ovary wall cells.

For the confirmation of infection in *R. sanguineus *females, a hemolymph test [20] was followed, where all the tested females of the control group presented hemocytes with normal morphology (Figure 1A) and the females of the infected group presented hemocytes with the cytoplasm filled with several bacteria (strongly stained) (Figure 1B). PCR showed that ten females from the control group did not react to the test and of ten females from the infected group, nine were positive in the test, confirming the infection by *R. rickettsii*.

### Histology of the ovaries in the Control Group

The ovaries of the females of the control group did not present any morphological variation, having as reference the pattern established by Oliveira *et al*. [19] in the first description of *R. sanguineus *ovary. Oocytes in several stages of development can be observed (Figure 1C-G), in addition to the pedicel cells connecting each oocyte to the ovary wall (Figure 1C-G).

### Histology of the ovaries in the Infected Group

**Figure 2:**
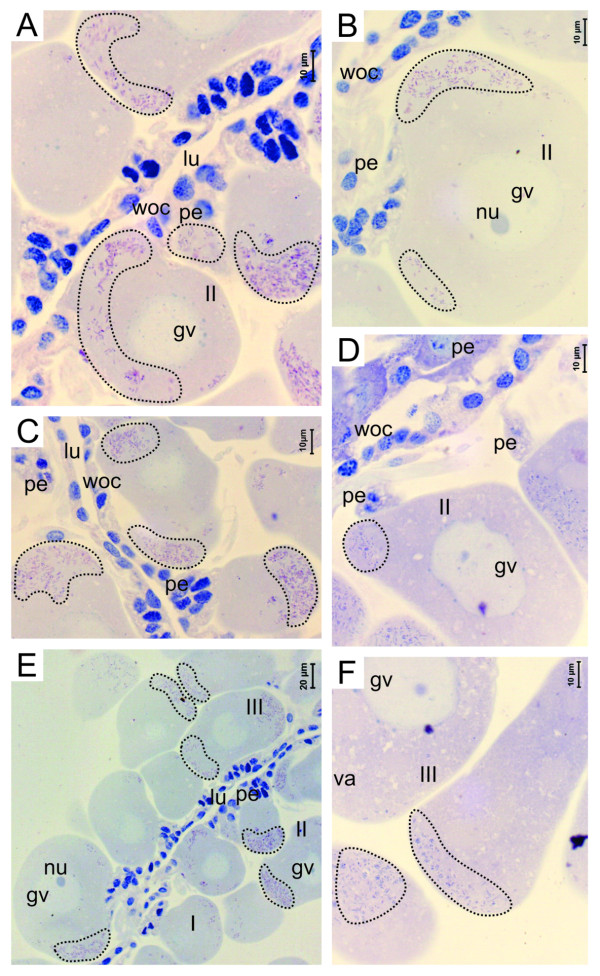
**Histological sections of the ovary of semi-engorged females infected by *R. rickettsii*, using Giemsa staining**. (A-F) Detail of the oocytes in developmental stages I, II and III with the presence of the bacterium *R. rickettsii *in the cytoplasm (dotted circle). ch, choriun; gv, germinative vesicle; lu, lumen; nu, nucleus; p, pedicel; woc, ovary wall cells.

The ovaries of the semi-engorged infected females contained oocytes in all stages of development. Although *R. rickettsii *has been detected in the oocytes in all stages of development they are more frequently found in the oocytes II and III (Figure 2A, B, D and 2F) and are most commonly located in the periphery of the oocyte (Figure 2A-F). It was also observed that the highest concentration of bacteria is located in the pole of the oocyte adjacent to the pedicel cells (Figure 2B and 2D), which were also colonized by the bacteria, although with fewer bacteria than found in the oocytes. Many oocytes with altered morphology were observed (Figure 2C and 2F) in contrast to oocytes from the control group. No *R. rickettsii *were observed in the cells of the ovary's wall.

**Figure 3:**
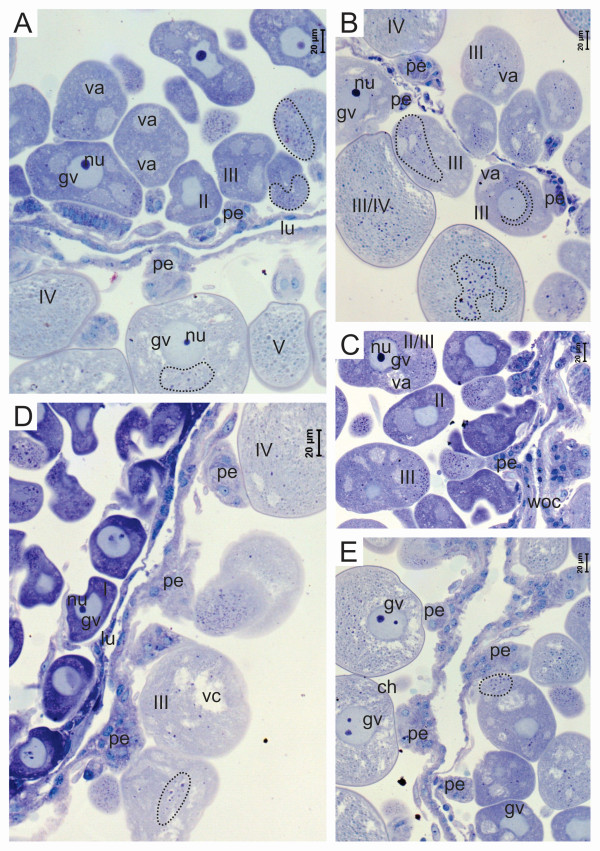
**Histological sections of the ovary of engorged females infected by *R. rickettsia*, stained using Giemsa**. (A-E) Detail of oocytes in all the stages of development with the presence of the bacterium *R. rickettsii *in the cytoplasm (dotted circle). ch, chorion; gv, germinal vesicle; lu, lumen; nu, nucleus; p, pedicel; woc, ovarian wall cells.

**Figure 4:**
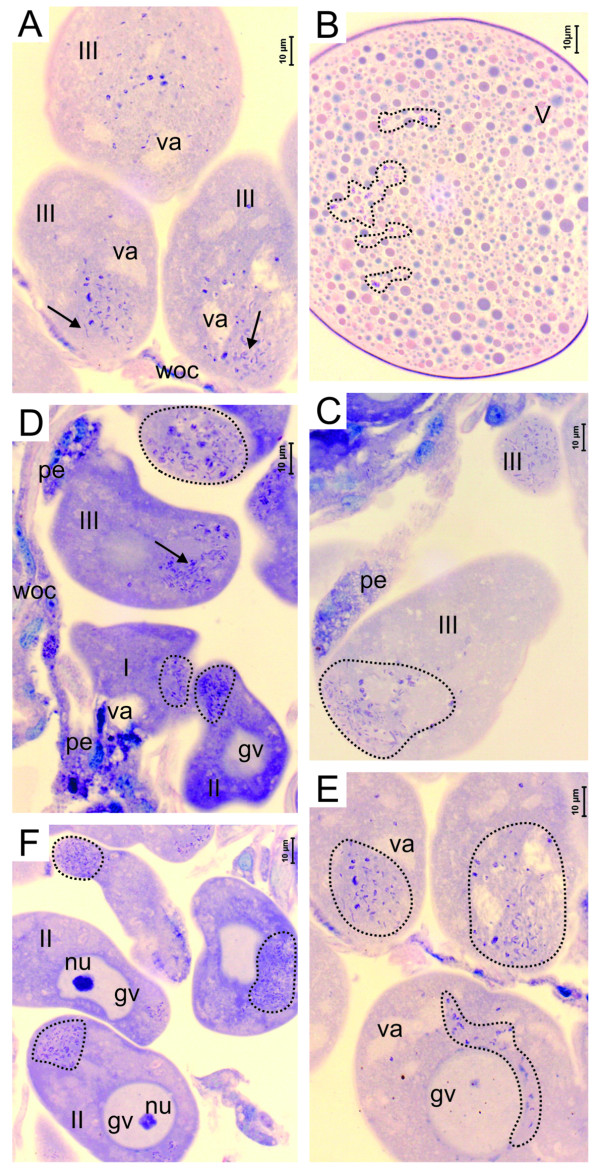
**Histological sections of a Giemsa-stained ovary of an engorged female infected by *R. rickettsii***. (A-E) Detail of the oocytes in all development stages with the presence of the bacterium *R. rickettsii *in the cytoplasm (dotted circle). (B) Detail of the oocyte V with *R. rickettsia *in its interior. ch, choriun; gv, germinative vesicle; lu, lumen; nu, nucleus; p, pedicel; woc, ovary wall cells; arrow, bacterium *R. rickettsia*.

Oocytes in the five stages of development were identified in the ovaries of engorged females (Figure 3 and 4). Infection by *R. rickettsii *was observed in the oocytes in all the stages of development (Figure 3B, 4B-D). The distribution of *R. rickettsii *in the oocytes was not homogeneous; in the semi-engorged females, groups of bacteria are most commonly located in the periphery of the oocytes (Figure 3A, 4C, D and 4F). In some cases bacteria are grouped around the germinal vesicle (Figure 3A and 4E).

The pedicel cells are also parasitized by *R. rickettsia *in semi-engorged and engorged females; with fewer numbers of bacteria in the cells of semi-engorged females (Figure 3A-E, 4C-D). As with the semi-engorged females, in engorged females the cells of the ovary wall were not infected with *R. rickettsii*.

## Discussion

The present study brings morphological data of the occurrence of transstadial and transovarial transmission of *R. rickettsii *in the dog tick *R. sanguineus *through histological analysis of the ovaries of semi-engorged and fully engorged females. The analysis of the results demonstrated that the infection provoked in the guinea pigs by adult *R. sanguineus *caused characteristic signs of BSF, as demonstrated by Mancini *et al*. [21], i.e., the animals presented temperature equal or higher than 40°C, in addition to scrotal lesion, with death of one individual. These results demonstrate the effective transstadial transmission in *R. sanguineus *when infected in the phase of nymph, corroborating the results obtained by Parker *et al*. [22] and Piranda *et al*. [23]. According to Burgdorfer & Brinton [24], the successful transovarial transmission depends primarily on the degree of rickettsial development in ovaries tissues. In this study *R. rickettsii *was observed in the oocytes in almost all of the stages of development, both in semi-engorged and in fully engorged females, corroborating Burgdorfer [25] and, Burgdorfer and Brinton [24] studying in the tick *Dermacentor andersoni*. Furthermore, Piranda *et al*. [23] using a PCR technique reported the transovarial transmission of *R. rickettsii *in *R. sanguineus*, also infected in nymph phase

The histological analysis of the ovaries of *R. sanguineus *infected females confirmed the presence of the bacterium, and indicated that the infection can interfere negatively in the process of reproduction of the ticks, as alterations were detected both in the shape and in the cell structure of the oocytes, which contained bacteria mainly in the fully engorged females - the ones that would be ready for oviposition. This supports the theory of Macdade and Newhouse [26], which postulates that the relationship between *R. rickettsii *and ticks is not always of perfect symbiosis; i.e., in some cases the microorganisms could harm the tick's organs causing damage to threaten its survival. Therefore, the fact that the oocytes of *R. sanguineus *infected by *R. rickettsii *show morphological alterations could suggest that the bacterium, in addition to being harmful for the host, could also be harmful to the reproductive process of the infected females.

The preferred distribution of *R. rickettsii *in the oocyte cytoplasm was at the oocyte pole in direct contact with the pedicel cells. Its presence in these cells and its absence from the ovary wall cells could suggest that, in addition to the hemolymph, the bacteria use the pedicel cells as a route of entry to the oocytes environment. This may occur when the cells of the pedicel transfer lipid, protein and polysaccharide material to the interior of the oocytes, helping in the production of yolk [19]. The pedicel cells are in turn infected by the bacteria from the hemolymph, from where they extract material to be used in vitellogenesis.

## Conclusions

Studying the relationship between ticks and pathogens has been very important to understand the mechanisms of infection and transmission of these pathogens, as well as to elaborate efficient strategies to avoid the diseases caused by them. Therefore, the present study clearly demonstrated that the transstadial transmission of *R. rickettsii *in *R. sanguineus *is efficient when these are infected in the phase of nymph. Although there is transovarial transmission in the females derived from these infected nymphs, the bacteria interfere negatively in the reproductive process by altering the morphology of the oocytes.

## Methods

Adult females of *R. sanguineus *infected with *R. rickettsii *were used in this study, which was performed in the Laboratory of Parasitary Diseases of the Department of Preventive Veterinary Medicine and Animal Health - VPS, Veterinary Medicine and Zootechny College of USP - University of Sao Paulo, SP, Brazil, under the supervision of Prof. Dr. Marcelo Bahia Labruna.

Two phases of infestation were necessary for the experiment, in accordance to Piranda *et al*. [23]. In the first phase, six guinea pigs were divided in two groups, the control group with three individuals (C1, C2, C3) and the infected group also with three individuals (I1, I2, I3). In each guinea pig of the infected group a 3 mL solution containing brains (n°: 7/9/2009) and liver (n°: 1-16/03/09) of guinea pigs positive for *R. rickettsii *and brain-heart infusion (BHI) was inoculated intraperitoneally after asepsis in the abdominal region according to the protocol described by Labruna *et al*. [8]. The temperature of the guinea pigs was measured daily during the whole feeding period in order to confirm the infection. After the feeding period the fully engorged nymphs were placed in a biochemical oxygen demand (BOD) incubator at 27°C, remaining there for 48 days. During this period the nymphs completed ecdysis, reaching the adult phase.

In the second phase of infection, six other guinea pigs were used, being divided into two groups (control and infected). The individuals from the control group were infested by adult *R. sanguineus *from the control groups of the first feeding period, while the individuals from the infected group were infested by *R. sanguineus *adults from the infected group of the first feeding period. As in the first phase, the temperature of the guinea pigs was measured daily for confirmation of infection.

### Analysis of the ticks

Females of *R. sanguineus*, which had fed for 5 days and the fully engorged females were prepared for the hemolymph test to confirm the infection and the ovaries were removed to be processed for histology. Ten females from the control group and 10 from the infected group were kept in a freezer at -20°C, for the extraction of DNA and performance of PCR according to the protocol described by Labruna *et al*. [27], for the confirmation of infection by *R. rickettsii*.

### Hemolymph test

The performance of the test followed the protocol described by Burgdorfer [20], where the distal portion of one of the front legs of the ticks was cut with scissors and one or two drops of hemolymph were placed onto a glass slide previously cleaned and de-greased. The slides were then fixed at room temperature, stained by the method of Gimenez [28] and examined and photographed using a Leica photomicroscope, in the Histology Laboratory of the Biology Department of the Biosciences Institute of UNESP *campus *Rio Claro (SP), Brazil.

### Histological technique

For the performance of histological techniques the ovaries were removed and fixed in 4% paraformaldehyde and in 10% neutral-buffered formaldehyde solution (pH 7-7.4) and acetone in the proportion 9:1 for 1 hour at room temperature and for 30 minutes at 4°C. The material was then dehydrated in increasing concentrations of ethanol (70%, 80%, 90% and 95%), for 15 minutes each, transferred to embedding resin and sectioned with a microtome in 3 μm-thick sections, which were collected on glass slides, rehydrated in distilled water for 1 minute and stained with solution containing Giemsa (8 g), Glycerol (500 mL) and Methanol (buffered pH 6.8, 1000 mL), dissolved in a buffered solution of NaOH4 (1:50), for 40 minutes, and washed in buffer. After drying, the slides were diaphanized in xylol, mounted in synthetic Canada balsam and covered with a coverslip. The material was observed and photographed using a Leica photomicroscope in the Histology Laboratory of the Biology Department of the Biosciences Institute of UNESP *campus *Rio Claro (SP), Brazil.

## Competing interests

The authors declare that they have no competing interests.

## Authors' contributions

LFSC and JFS performed the infection of ticks with *R. rickettsii*, LFSC and PHN performed the histological analysis and wrote the manuscript, MBL and MICM supervised the research and the writing of the manuscript. All authors approved the final, version of the manuscript.
